# Er Miao San Attenuates Collagen-Induced Arthritis Mice by Regulating Gut Microbiota and Its Metabolites

**DOI:** 10.4014/jmb.2507.07054

**Published:** 2025-11-26

**Authors:** Shili Xu, Wenrui Su, Zhifang Qin, Zihua Xuan, Jiayu Wang, Jin Wang, Ran Tang, Jiahua Yin, Juan Liang, Xiaoyi Jia

**Affiliations:** 1 School of Pharmacy, Anhui University of Chinese Medicine, Hefei 230012, P.R. China; 2 Anhui Province Key Laboratory of Bioactive Natural Products, Hefei 230012, P.R. China

**Keywords:** Er Miao San, rheumatoid arthritis, gut microbiota

## Abstract

Dysbiosis of the gut microbiota plays a key role in the pathogenesis of rheumatoid arthritis (RA). However, it is still unclear whether the classic prescription Er Miao San (EMS) can exert therapeutic effects on RA by regulating the gut microbiota. In this study, we investigated whether EMS alleviates collagen-induced arthritis (CIA) by modulating the gut microbiota and its metabolites. We demonstrated that EMS significantly reduced arthritis severity, paw swelling, and systemic inflammation in CIA mice. In addition, 16S rRNA sequencing analysis revealed that EMS restored gut microbiota homeostasis, as evidenced by an increased abundance of Bacteroidetes, and a decreased Bacteroidetes/Firmicutes ratio. Crucially, antibiotic depletion of the gut microbiota abolished the protective effects of EMS, whereas fecal microbiota transplantation (FMT) from EMS-treated donors replicated its anti-arthritic efficacy, confirming the indispensable role of the microbiota. Measurement of short-chain fatty acids (SCFAs) further revealed a significant increase in the microbial metabolite butyrate following EMS treatment. Subsequent supplementation with sodium butyrate mimicked the therapeutic effects of EMS, ameliorating joint inflammation and cartilage damage. Mechanistically, butyrate enhanced the expression of intestinal tight junction proteins (ZO-1 and occludin), thereby restoring intestinal barrier integrity. Collectively, our results demonstrate that EMS exerts its anti-arthritic effects by modulating the gut microbiota-butyrate-intestinal barrier axis, highlighting the critical value of microbial metabolites in RA treatment. This study provides novel insights into the mechanism of EMS and suggests the therapeutic potential of butyrate for RA.

## Introduction

Rheumatoid arthritis (RA) is a chronic autoimmune disease characterized by synovial inflammation [1, 2]. Increasing evidence suggests that the gut-joint immune axis has a main role in the pathogenesis of RA [3], and imbalance in the host gut microbiota is a key contributing factor [4, 5]. Dietary modifications or pharmacological interventions are known to positively reshape the gut microbiota composition [6-8], which suggests the therapeutic potential of microbiota modulation for RA treatment. Meanwhile, traditional Chinese medicine (TCM) has demonstrated efficacy in treating various illnesses and is gaining attention. However, the complex composition and pharmacological mechanisms of TCM formulations require further investigation. Emerging evidence indicates that some of these medicines can significantly alleviate RA by modulating gut microbiota and its metabolites [9, 10], suggesting a promising avenue for research.

Er Miao San (EMS) is a TCM formulation with demonstrated anti-inflammatory and immunomodulatory properties. Our previous research has shown that it exerts anti-RA effects by modulating dendritic cells, T cells, and macrophage polarization, as well as inhibiting the Wnt/β-catenin pathway [11, 12]. The gut-targeting potential of berberine, a key component of EMS, is well known [13]. Furthermore, some herbal compounds have been linked to the gut microbial short-chain fatty acids (SCFAs)-immune-modulation axis [14]. However, it remains unclear whether the anti-arthritic effect of the entire EMS formula is superior to that of its individual components by being mediated through this mechanism.

Therefore, in this study we investigated whether EMS exerts its therapeutic effects against RA, specifically through remodeling the gut microbiome and promoting the production of beneficial metabolites, to provide direct evidence for its gut-mediated mechanisms of action.

## Materials and Methods

### Plant Materials

EMS is composed of Atractylodis Rhizoma (AR), also known as the rhizome of *Atractylodes lancea* (Thunb.) DC. (Compositae), and Phellodendri Cortex (PC), also known as the bark of *Phellodendron chinensis* Schneid (Rutaceae). All herbs were purchased from Bozhou Qiaocheng Pharmaceutical Co., Ltd. (China). The samples were identified by Dr. Liu SJ (School of Pharmacy, Anhui University of Chinese Medicine), and one specimen of each (ID: EMS-22-01) was preserved in the Herbarium of Pharmacy, School of Pharmacy, Anhui University of Chinese Medicine (China). The EMS was extracted with reference to the method in the published literature [15, 16].

### Reagents and Antibodies

Antibodies for occludin (Cat. 66378-1g); ZO-1 (Cat. 21773-1-AP); β-actin (Cat. 66009-1-Ig) (Proteintech, China); secondary antibodies (Cat. SA00001-1 and Cat. SA00001-2) (Proteintech); ELISA kits for mice TNF-α (Cat. EK282/4); IL-6 (Cat. EK206/3); IL-10 (Cat. EK210/4); IL-17A (Cat. EK217/2) (MultiSciences Biotech, China); LPS ELISA kit (Cat. ML002796) (Enzyme Linked Biotechnology, China); sodium butyrate (Cat. C15417275)(McLean Biochemical Technology, China); gentamicin sulfate (Cat. 1453GR005); ampicillin sodium (Cat. 1146GR005); vancomycin hydrochloride (Cat. 1161GR001); streptomycin sulfate (Cat. 1297GR005) (BioFROXX Biochemical Technology, China).

### Animals

Eight-week-old male DBA/1 mice were procured from Jiangsu GemPharmatech Co., Ltd., China (license SCXK2023-0009) and accommodated at the Laboratory Animal Center, Anhui University of Chinese Medicine. The mice were kept in cages measuring 130 × 200 × 300 mm, with a maximum of five mice per cage. Housing conditions followed standard laboratory protocols, including a controlled temperature of 22°C–26°C and a 12-h light-dark cycle. The Experimental Animal Ethics Committee of Anhui University of Chinese Medicine approved all experiments (No. AHUCM-mouse-2023134).

### Establishment of Collagen-Induced Arthritis (CIA) Model and Experimental Grouping

A stock solution of Type II Collagen from Chicken (CII) was prepared by dissolving 10 mg of lyophilized CII in 5 ml of 0.05 molar acetic acid at 4°C with gentle stirring overnight. On day 0, CII was mixed with complete Freund's adjuvant (CFA) in equal volumes to achieve a concentration of 4 mg/ml. Mice were subcutaneously injected with 150 μl of this suspension at the dorsal region. On day 21, a booster immunization consisting of 100 μl of CII-CFA suspension was administered.

Following the successful induction of CIA, mice were stratified according to initial arthritis scores and body weight, and then randomly assigned to experimental groups using a random number table. The mice were divided into three groups (*n* = 6 per group): Normal (N), CIA, and EMS (2 g/kg). Starting on day 28, the EMS group received daily oral gavage of EMS for 28 days. The CIA group received an equivalent volume of vehicle daily. The mice were sacrificed after the experiment, and their serum, joint tissues and intestinal contents were collected for further study.

### Antibiotic Cocktail (ATBX) Treatment and Fecal Microbiota Transplantation (FMT)

The ATBX contained 0.5 g/l ampicillin sodium, 1 g/l streptomycin sulfate, 0.5 g/l vancomycin hydrochloride, and 1 g/l gentamicin sulfate. CIA mice in the ATBX+EMS group received the ATBX in drinking water for 7 days from day 21, followed by EMS (2 g/kg) administered via daily oral gavage from day 28 for 28 days.

For FMT, recipient mice received the ATBX for 7 days prior to model induction to deplete gut microbiota. Feces (100 mg) from the donor mice was resuspended in 1 ml of sterile saline. After centrifugation at 4°C at 10,000 ×*g* for 10 min, the supernatant containing bacteria was used as transplant material. Fresh transplant material was prepared within 10 min before oral gavage to maintain the activity of fecal bacteria. Subsequently, starting concurrently with CIA induction, these mice underwent daily FMT via oral gavage for 57 consecutive days. The FMT-CIA group received microbiota from CIA donor mice, while the FMT-EMS group was administered microbiota from donors treated with EMS (2 g/kg).

### SB Treatment in CIA Mice

After booster immunization on day 21, CIA mice were randomly divided into three groups (*n* = 6 per group): CIA, EMS (2 g/kg), and SB (200 mg/kg). Starting on day 28, the EMS and SB groups were administered their respective treatments by daily oral gavage for 28 days.

### Evaluation of Arthritis

Two independent observers, who were unaware of the treatment regimen, assessed the severity of CIA. Arthritis severity was evaluated every three days based on the arthritis index and the number of swollen joints. The arthritis index scoring criteria are shown in Table 1. Joint swelling counts were assessed as follows: one ankle (or wrist) and five toe joints per paw per mouse were counted using a vernier caliper.

### Hematoxylin and Eosin (H&E) Staining

The ankle joints of mice were dissected, fixed in 4% paraformaldehyde, decalcified, and embedded in paraffin. The paraffin-embedded tissues were then sectioned, stained with H&E, and observed under a light microscope.

### Knee Blood Flow Signal Detection

The knee joints of mice were shaved to remove hair. Mice in each group were anesthetized using a gas anesthesia machine with isoflurane. A medical ultrasound coupling agent was applied to the knee joints, and blood flow signals were examined using an ultrasound probe.

### 16S rRNA Gene Sequencing and Profiling

Fecal samples from mice were collected on day 57 and stored at -80°C. High-throughput sequencing of 16S rRNA was performed by Majorbio Tech Co., Ltd., (China). Differences in gut microbiota among the normal (N), CIA, and EMS groups were evaluated using principal coordinates analysis (PCoA). Dominant microbiota in different groups were identified through linear discriminant analysis and effect size (LEfSe).

### Detection of SCFA Content

SCFAs in cecal content samples were measured via gas chromatography (GC) after derivatization. Samples were accurately weighed using an analytical balance and placed in 1.5 ml centrifuge tubes. Ultrapure water was added at a ratio of 1:2.5, vortexed for 5 min to mix thoroughly, and centrifuged at 15,000 ×*g* for 10 min. The supernatant was collected, mixed with 25% metaphosphoric acid at a ratio of 5:1, acidified in an ice bath for 1 h, and centrifuged again at 15,000 ×*g* for 10 min. Afterward, 1 mL of the supernatant was transferred into an injection vial for GC analysis following derivatization.

### Enzyme-Linked Immunosorbent Assay (ELISA)

Blood samples were collected after an overnight fast in tubes containing disodium ethylenediaminetetraacetate dihydrate (Na_2_-EDTA) at a concentration of 1 mg/ml. Serum TNF-α, IL-6, IL-17A and IL-10 levels were measured using ELISA kits, following the manufacturers’ protocols.

### Western Blot

Western blot was employed to examine the protein expression of occludin and ZO-1. Tissues were disrupted in RIPA buffer (Beyotime Biotechnology, China) supplemented with PMSF (Beyotime). Protein lysates were preserved in 1.5 ml tubes at -20°C until examination. The proteins were separated using SDS-PAGE and shifted to PVDF membranes. The membranes were exposed overnight to primary antibodies, and subsequently cultivated with HRP-conjugated secondary antibodies. Signals were detected using enhanced chemiluminescence (ECL) substrates (Sparklade, China). Protein expression levels were standardized to endogenous β-actin levels.

### Data and Statistical Analysis

Statistical analysis was performed using SPSS 23.0 (SPSS, USA), and graphs were generated with GraphPad Prism 8.0 (GraphPad, USA). Continuous variables following a normal distribution were presented as mean ± SD ($\bar{\text{x}}$ ± s). For comparisons between two groups whose data conformed to normal distribution, the *t*-test was adopted, whereas one-way ANOVA was employed for comparisons among multiple groups. In the case of non-normally distributed data or groups with unequal variances, the Kruskal-Wallis H test was resorted to. A significance level of 0.05 was set, and the Bonferroni method was used to adjust multiple comparisons so as to take potential errors into consideration.

## Results

### EMS Ameliorated Arthritis Severity and Systemic Inflammation in CIA Mice

**Fig. 1:**
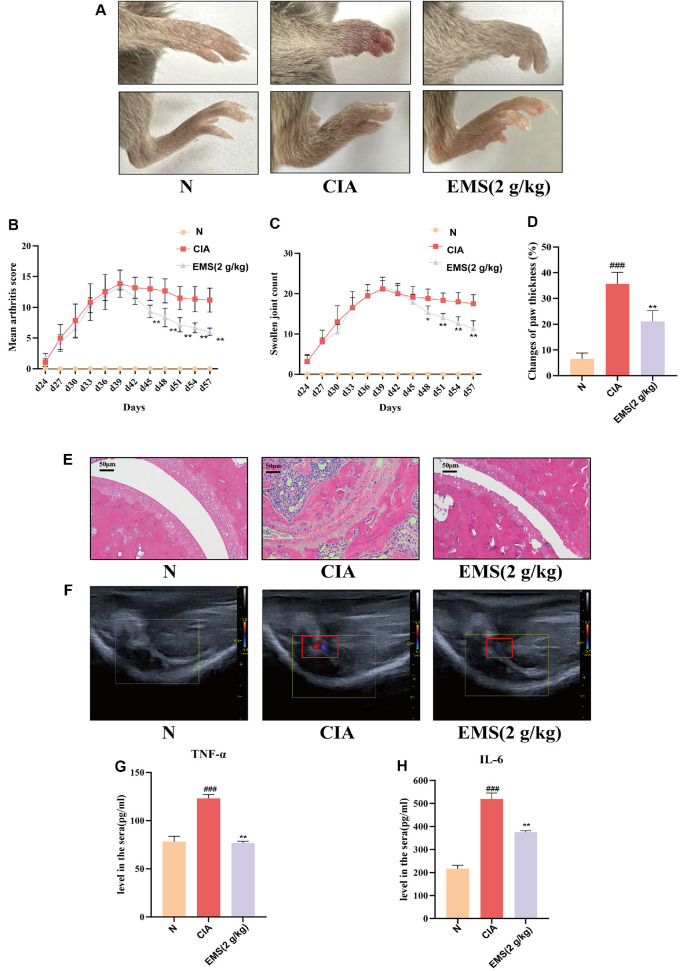
EMS alleviates arthritis severity and systemic inflammation in CIA mice. (**A**) Representative photographs of paw swelling in DBA/1 mice (*n* = 6). (**B**) Arthritis index (*n* = 6). (**C**) Counts of swollen joints (*n* = 6). (**D**) Growth rate of paw thickness (*n* = 6). (**E**) HE staining of the ankle joint (×20 magnification) Scale bar: 50 μm (*n* = 3). (**F**) Knee blood flow signals (*n* = 3). (G-H) Expression levels of TNF-α and IL-6 in the serum of CIA mice. N means Normal group, CIA means CIA group, EMS (2 g/kg) means EMS group (*n* = 4). Data are presented as the mean ± SD. *p < 0.05, **p < 0.01 vs. CIA group; ^##^*p* < 0.01, ^###^*p* < 0.001 vs. N group.

To evaluate the therapeutic effect of EMS on RA, a CIA mouse model was established. Mice were treated daily with EMS (2 g/kg) via oral gavage. EMS treatment significantly alleviated arthritis severity, as evidenced by reduced paw redness/swelling, lower arthritis index, fewer swollen joints (Fig. 1A-1C), and a suppressed paw thickness growth rate (Fig. 1D). Histopathological analysis revealed that EMS inhibited synovial hyperplasia, reduced inflammatory cell infiltration, and ameliorated cartilage erosion in the ankle joints of mice (Fig. 1E). Ultrasound imaging showed increased blood flow in the knee joints of CIA mice, which was significantly reduced after EMS treatment (Fig. 1F). Furthermore, EMS administration resulted in a significant reduction in serum TNF-α and IL-6 levels (Fig. 1G and 1H).

### EMS Restored Gut Microbiome Homeostasis in CIA Mice

**Fig. 2:**
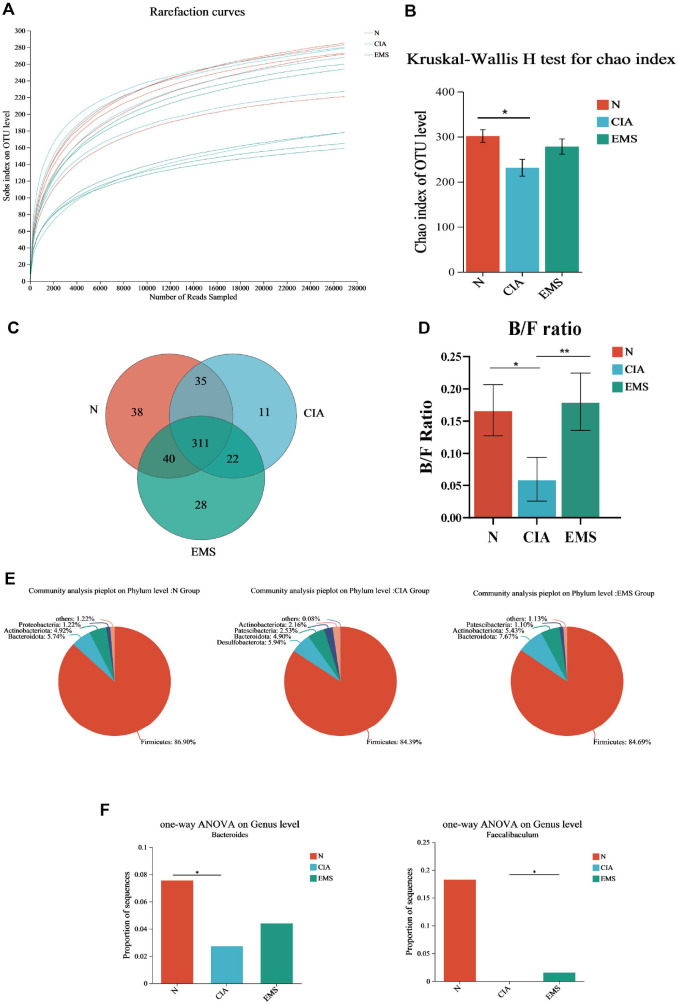
EMS modulates gut microbiota composition and diversity in CIA mice. (**A**) Sobs index dilution curve (*n* = 5). (**B**) Chao index (*n* = 5). (**C**) Degree of overlap of OTUs of gut microbes in three groups of mice (*n* = 5). (**D**) Bacteroidota/Firmicutes ratio in the intestines of three groups of mice (*n* = 5). (**E**) Percentage of phylum level flora in three groups of mice (*n* = 5). (**F**) Abundance of *Bacteroides* and *Faecalibacterium* in mice intestine (*n* = 5). *p <0.05, **p <0.01.

Based on the systemic immunomodulatory effects observed, we hypothesized that the gut-joint axis might be involved in the mechanism of EMS. To test this, we characterized the gut microbial community by analyzing cecal contents from Normal (N), CIA, and EMS (2 g/kg) mice using 16S rRNA sequencing. Analysis of α-diversity revealed significant alterations among groups. Adequate sequencing depth was confirmed by plateauing Sobs index curves (Fig. 2A). EMS significantly increased the Chao1 index in CIA mice (Fig. 2B). Moreover, there was a greater number of shared operational taxonomic units (OTUs) between the N and EMS groups than between the N and CIA groups, indicating that EMS administration brought the gut microbiota of CIA mice closer to that of the Normal group (Fig. 2C). At the phylum level, EMS increased Bacteroidota levels while significantly decreasing the Bacteroidota/Firmicutes (B/F) ratio in the intestinal tracts of CIA mice compared with the Normal group (Fig. 2D). Moreover, EMS treatment elevated the abundance of Bacteroidota (Fig. 2E). At the genus level, the abundances of *Bacteroides* and *Faecalibacterium* were significantly reduced in the CIA group. EMS restored the abundance of both genera to levels comparable to those in the Normal group (Fig. 2F).

**Fig. 3:**
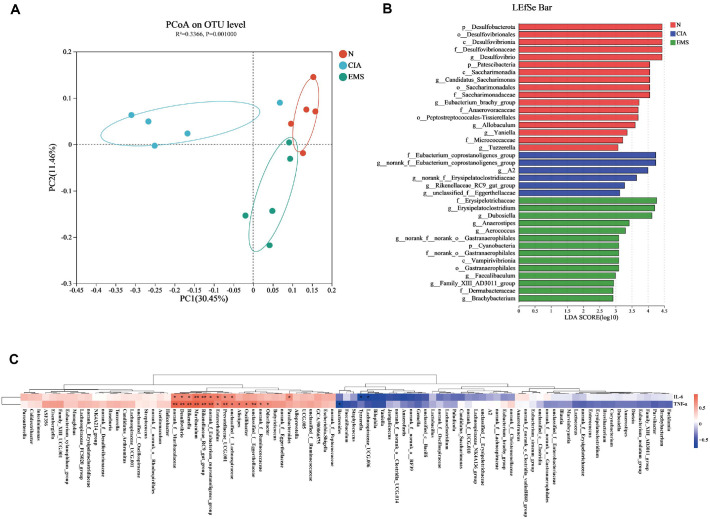
EMS restores gut microbial community structure and reveals correlations with inflammation. (**A**) PCoA analysis based on unweighted_unifrac (*n* = 5). (**B**) LEfSe analysis of gut microbes in mice (*n* = 5). (**C**) Correlation analysis of microbe abundance with serum inflammatory cytokines (*n* = 5). *p <0.05, **p <0.01.

PCoA revealed clear separation between the Normal and CIA groups, while the EMS group exhibited a microbial structure closer to that of normal mice (Fig. 3A). LEfSe analysis was used to compare the cecal microbiota composition among the three groups. The linear discriminant analysis (LDA) score histogram (Fig. 3B) depicted the distribution of predominant bacteria in each group: 17 in the Normal group, 6 in the CIA group, and 14 in the EMS group. To further understand the relationship between altered microorganisms and serum TNF-α and IL-6 levels, Spearman correlation analysis was conducted. It revealed positive correlations between TNF-α and IL-6 levels and multiple genera, including unclassified_*f__Lachnospiraceae*, *Prevotellaceae_UCG.001*, *Enterorhabdus*, *norank_f__Eubacterium_coprostanoligenes_group*, *Rikenellaceae_RC9_gut_group*, *Muribaculum*, *Rikenella*, *Desulfovibrio*, and *norank_f__Muribaculaceae* (Fig. 3C).

### The Protective Effect of EMS on CIA Depended on the Gut Microbiota

**Fig. 4:**
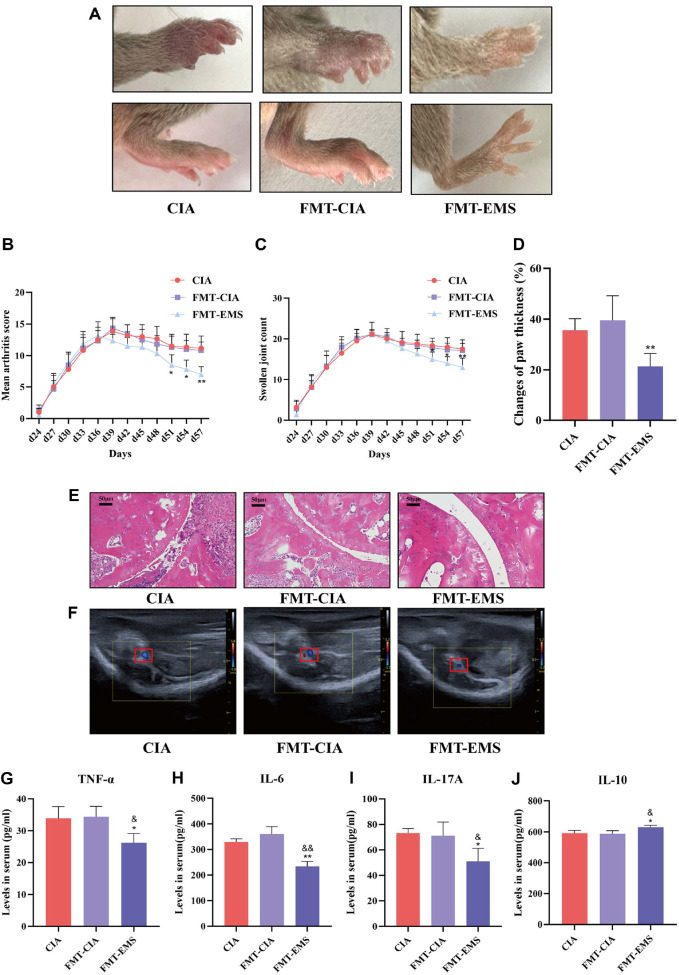
EMS microbiota transplantation ameliorates arthritis in recipient CIA mice. (**A**) Representative plots of paw swelling in each group of mice (*n* = 6). (**B**) Arthritis index (*n* = 6). (**C**) Swollen joint count (*n* = 6). (**D**) Growth rate of paw thickness (*n* = 6). (**E**) HE staining of the ankle joint (HE, ×200 magnification) Scale bar: 50 μm (*n* = 3). (**F**) Knee blood flow signals (*n* = 3). (**G-J**) Levels of TNF-α, IL-6, IL-10, and IL-17A in the serum of CIA mice (*n* = 4). Data are presented as the mean ± SD. **p <0.01 vs. FMT-CIA group, ^&^*p* <0.05, ^&&^*p* <0.01 vs. CIA group.

To determine whether the gut microbiota mediates EMS's anti-arthritic effects, mice were treated with ATBX to deplete gut microbes prior to EMS administration. Ablation of the gut microbiota markedly attenuated the therapeutic benefits of EMS on arthritis severity (Fig. S2A–S2F), indicating that an intact gut microbiota is essential for the efficacy of EMS. Compared with the FMT-CIA group, the FMT-EMS group showed significantly alleviated joint inflammation and markedly improved pathological changes in the ankle joint (Fig. 4A-4E). Ultrasound images showed that the blood flow in the knee joint was significantly decreased in the FMT-EMS group (Fig. 4F). Additionally, the levels of pro-inflammatory cytokines (TNF-α, IL-6 and IL-17A) were lower in the FMT-EMS group, while the expression level of the anti-inflammatory cytokine IL-10 was markedly higher than in both the FMT-CIA and the CIA groups (Fig. 4G–4J). Altogether, these results demonstrate that an intact gut microbiota is required for the protective effect of EMS on CIA mice.

### EMS Altered the Levels of Gut Microbiota Metabolite SCFAs in CIA Mice

**Fig. 5:**
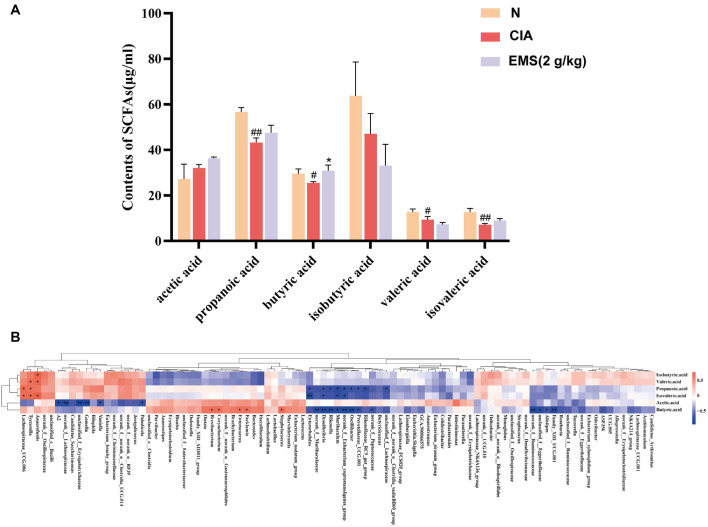
EMS alters the levels of gut microbiota metabolite SCFAs in CIA mice. (**A**) GC detection of acetic acid, propionic acid, butyric acid, isobutyric acid, valeric acid, and isovaleric acid levels in the contents of the cecum of each group of mice (*n* = 5). (**B**) Correlation analysis of microbe abundance with SCFAs levels (*n* = 5). Data are presented as the mean ± SD. *p <0.05 vs. CIA group, ^#^*p* <0.05 vs. N group, ^##^*p* <0.01 vs. N group.

Literature reports suggest that certain *Bacteroides* species can ferment fibers into SCFAs, which have immune-modulating and anti-inflammatory effects. GC analysis revealed that CIA group had significantly lower cecal levels of propionate, butyrate, valerate, and isovalerate than Normal group. EMS treatment selectively restored butyrate levels to normal, but not the other SCFAs (Fig. 5A). The levels of acetate and isobutyrate showed no statistically significant differences among the groups. This targeted restoration highlights butyrate as the pivotal metabolite mediating the gut-level effects of EMS. Spearman correlation analysis identified 29 genera significantly associated with SCFAs levels. Genera positively correlated with butyrate included *Staphylococcus*, *Brevibacterium*, *Corynebacterium*, *Enterococcus*, and *Facklamia*. In contrast, genera negatively correlated with butyrate included *Alistipes*, *Family_XIII_UCG.001*, *norank_f__Peptococcaceae*, *Oscillibacter*, and *Prevotellaceae_UCG.001* (Fig. 5B). These findings suggest that EMS influences SCFA concentrations in the gut metabolites of CIA mice by altering their microbiota composition. Notably, the significant increase in butyrate levels in the EMS group highlights its potential as a key metabolite for treating RA via EMS.

### SB Mediated the Therapeutic Effects of EMS in CIA Mice

**Fig. 6:**
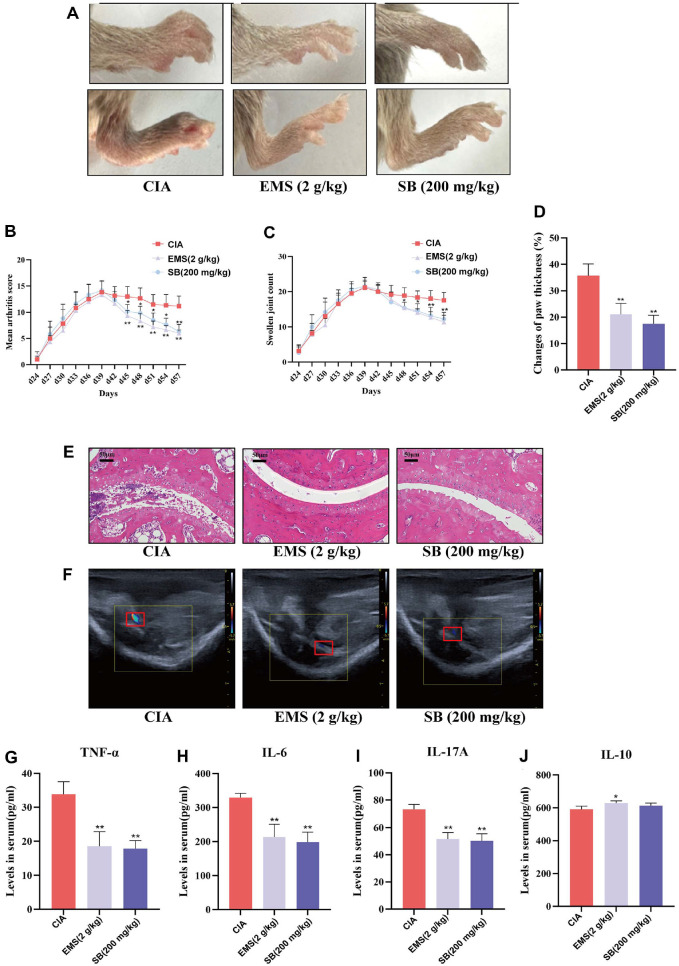
Butyrate mediates the therapeutic effects of EMS in CIA mice. (**A**) Representative graph of paw swelling in DBA/1 mice (*n* = 6). (**B**) Arthritis index (*n* = 6). (**C**) Swollen joint count (*n* = 6). (**D**) Growth rate of paw thickness (*n* = 6). (**E**) HE staining of the ankle joint (HE, ×200 magnification) Scale bar: 50 μm (*n* = 3). (**F**) Knee blood flow signals (*n* = 3). (**G-J**) Expression levels of TNF-α, IL-6, IL-17A, and IL-10 in the serum of CIA mice (*n* = 4). Data are presented as the mean ± SD. *p <0.05, **p <0.01 vs. CIA group.

To further validate the therapeutic effects of SB on CIA mice, a reconstructed CIA model was administered SB (200 mg/kg) via oral gavage (Fig. 6A). The treatment resulted in a significant reduction in swollen joint counts, arthritis index, and paw thickness growth rate in the SB group (Fig. 6B-6D). HE staining demonstrated attenuated cellular infiltration and reduced cartilage destruction in the ankle joints of SB-treated mice (Fig. 6E). Ankle blood flow was significantly lower in the SB group than in the CIA group (Fig. 6F). ELISA results showed significantly reduced levels of TNF-α, IL-6, and IL-17A in the SB group. Additionally, a marked elevation in IL-10 levels was observed in the EMS group (Fig. 6G-6J).

### EMS Alleviated CIA by Promoting SB-Dependent Restoration of the Intestinal Barrier

**Fig. 7:**
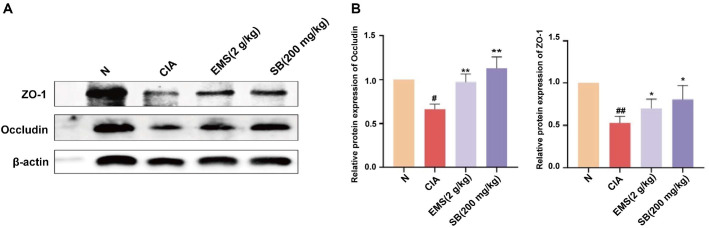
EMS alleviates CIA by promoting butyrate-dependent restoration of the intestinal barrier. (**A-B**) Western blot detection of small-intestinal tissue tight junction proteins (*n* = 6). Data are presented as the mean ± SD. *p <0.05, **p <0.01 vs. Normal group, ^#^*p* <0.05, ^##^*p* <0.01 vs. CIA group.

To investigate the mechanism of action of SB in the treatment of RA, western blot analysis of proteins extracted from small-intestinal tissues confirmed a significant reduction in ZO-1 and occludin protein levels in the CIA group, while a marked increase in these protein levels was observed in the SB group (Fig. 7A and 7B). These findings suggest that SB enhances the expression of tight junction proteins in the small intestine, helping restore the integrity of the intestinal barrier in CIA mice. This indicates that EMS may elevate butyrate levels, promoting intestinal barrier repair and potentially alleviating arthritis symptoms in CIA mice.

## Discussion

The pathogenesis of RA involves complex genetic, immune, and environmental interactions, with the gut microbiome emerging as a pivotal modulator [17-19]. Within this context, gut dysbiosis, particularly a reduction in beneficial SCFA-producing bacteria, has been implicated in RA development [20, 21]. However, a major challenge in the field has been establishing causality beyond mere correlation between microbial alterations and RA [22, 23]. In this study, we demonstrate that EMS alleviates RA by restoring the gut microbial homeostasis, thereby establishing a causal chain in which specific microbial changes enhance butyrate-mediated barrier restoration, ultimately leading to joint improvement.[Fig F8]

Our initial findings confirmed the therapeutic potential of EMS in the CIA model, showing significant alleviation of arthritis severity and suppression of pro-inflammatory cytokines. Given these systemic improvements, we hypothesized that the gut microbiota might be a key mediator. Analysis of microbial composition revealed that EMS alleviated arthritis by restoring gut microbial homeostasis [24]. This restoration was characterized by a selective enrichment of key butyrate-producing genera, particularly *Bacteroides* and *Faecalibacterium*—the former being closely associated with butyrate metabolic pathways and the latter a major producer with recognized anti-inflammatory properties [25, 26]. This targeted remodeling suggests a potentially distinct mechanism of action.

To unequivocally establish causality, we performed critical interventional experiments. Antibiotic depletion of the gut microbiota abolished the therapeutic benefits of EMS, confirming that an intact microbiome is necessary. More importantly, FMT from EMS-treated donors conferred protection to recipient mice, demonstrating that the EMS-modified microbiota is sufficient to elicit the therapeutic effect. This level of causal validation constitutes a key advance beyond associative studies and firmly establishes the gut microbiota as a central mediator of the effect of EMS.

We next sought to identify the key microbial metabolites responsible for these effects. Consistent with the enrichment of butyrogenic bacteria, EMS treatment led to a marked and selective increase in cecal butyrate levels, while the levels of other SCFAs, like propionate and valerate, remained unchanged. To directly test the functional role of this specific increase, we performed supplementation experiments with SB. These experiments confirmed that SB alone mimicked key therapeutic effects of EMS, including the amelioration of arthritis, inhibition of pro-inflammatory cytokines (IL-6, TNF-α, and IL-17A), and crucially, the restoration of intestinal barrier integrity (ZO-1 and occludin). The suppression of IL-17A implicates the Th17 pathway as one immune mechanism [11, 27], while the restoration of tight junction proteins directly links butyrate to the preservation of the gut barrier, providing strong experimental support for the "gut-joint axis" theory [28, 29].

While this identified pathway is central, it likely operates within a broader context of microbial remodeling. The efficacy of FMT from EMS-treated donors suggests that additional immunomodulatory metabolites, such as tryptophan derivatives or bile acids, may also contribute to the overall therapeutic outcome, and their exploration constitutes an important focus for future research. Therefore, our model positions butyrate-dependent gut barrier restoration as a clearly defined and essential pathway, while acknowledging potential parallel or synergistic mechanisms that may operate concurrently. Our interventional experiments, particularly the supplementation with SB, define a critical role for butyrate in mediating the therapeutic effects of EMS.

Compared to broad-spectrum traditional herbal formulations such as Wu-tou decoction, which operate through multiple components and targets, EMS exhibits highly precise targeting by focusing on the butyrate metabolic pathway, achieving dual modulation—from the structural enrichment of specific bacterial populations to the functional elevation of butyrate levels [9, 10]. Furthermore, unlike interventions such as berberine, for which only correlations between butyrate and therapeutic effects have been reported, we have provided a complete chain of causal evidence for the "gut microbiota–butyrate–efficacy" axis through antibiotic depletion and FMT experiments, thereby advancing the field from association to causation [13].

## Conclusion

In summary, our study has established a causal "gut microbiota-butyrate-gut barrier-joint" axis, wherein the elevation of butyrate constitutes a pivotal step linking microbial remodeling to the amelioration of joint inflammation. EMS may exert its therapeutic effect on RA by regulating this axis. The above findings highlight the significance of integrating microbiome analysis with causal intervention strategies. This approach helps to better understand the mechanism of action of complex phytomedicine, and provides a solid scientific foundation for future clinical applications.

## Supplemental Materials

Supplementary data for this paper are available on-line only at http://jmb.or.kr.



## Figures and Tables

**Fig. 8 F8:**
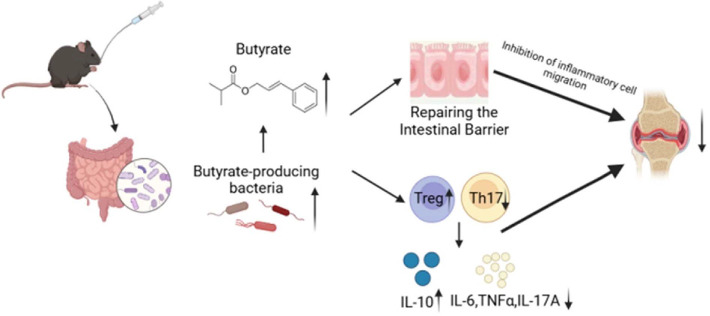
Schematic diagram of the mechanism by which EMS regulates the intestinal flora and its metabolites to ameliorate CIA in mice.

**Table 1:** Arthritis Index Scoring Criteria.

Score	Symptom presentation
0	No redness or swelling
1	Mild swelling of the metatarsophalangeal joint
2	Swelling of the metatarsophalangeal joint and plantar surface
3	Swelling of the entire paw below the ankle
4	Swelling of the entire paw including the ankle
