# Supplementary material for: Er Miao San Attenuates Collagen-Induced Arthritis Mice by Regulating Gut Microbiota and Its Metabolites
Source: J Microbiol Biotechnol. 2025 Nov 26;35:e2507054. doi: 10.4014/jmb.2507.07054 (PMC12685576; doi:10.4014/jmb.2507.07054)
Supplement: Supplementary file 1 [file jmb-35-e2507054-supple.pdf]

J. Microbiol. Biotechnol. 2025. 35: e2507054
https://doi.org/10.4014/jmb.2507.07054

Supplementary Figures

A

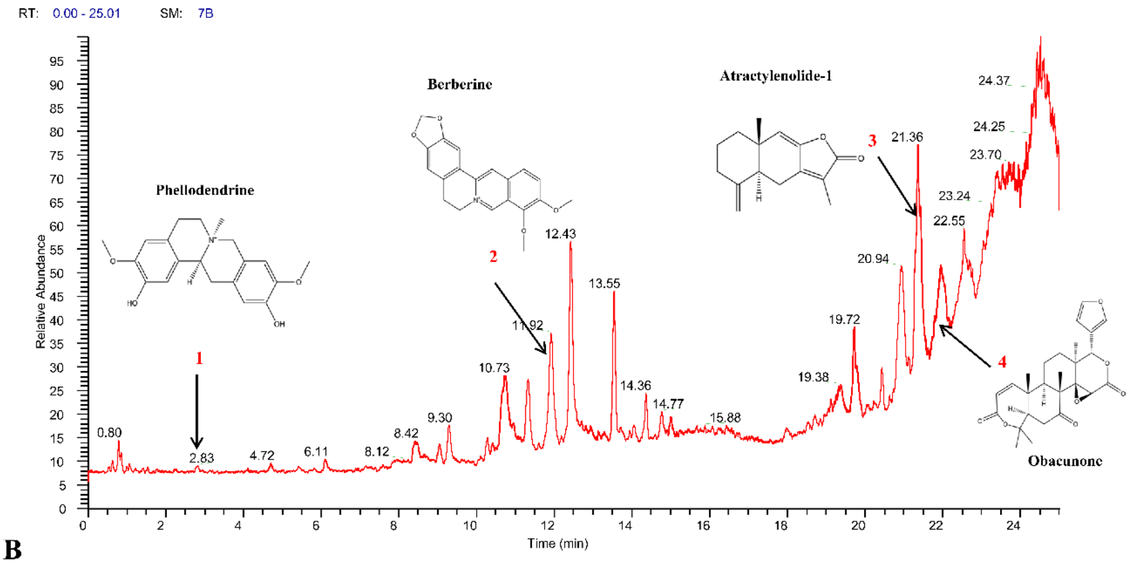

B

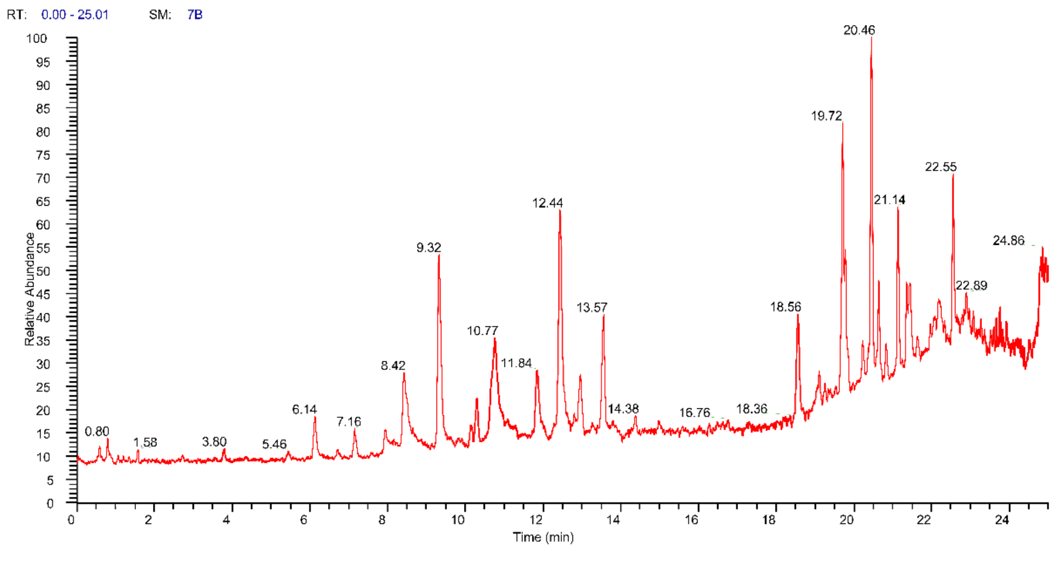

Fig. S1. Identification of active compounds of EMS with UPLC- QE-Orbitrap-MS

(A) The total ion chromatography of EMS on positive. **(B)** The total ion chromatography of EMS on negative.

Comprehensive profiling was conducted using the UPLC-QE-Orbitrap-MS to qualitatively analyze the composition of the ethyl acetate extract of EMS. This analysis identified several major bioactive compounds, including phellodendrine, berberine, atractylenolide-1 and obacunone. (Fig. S1A-B)

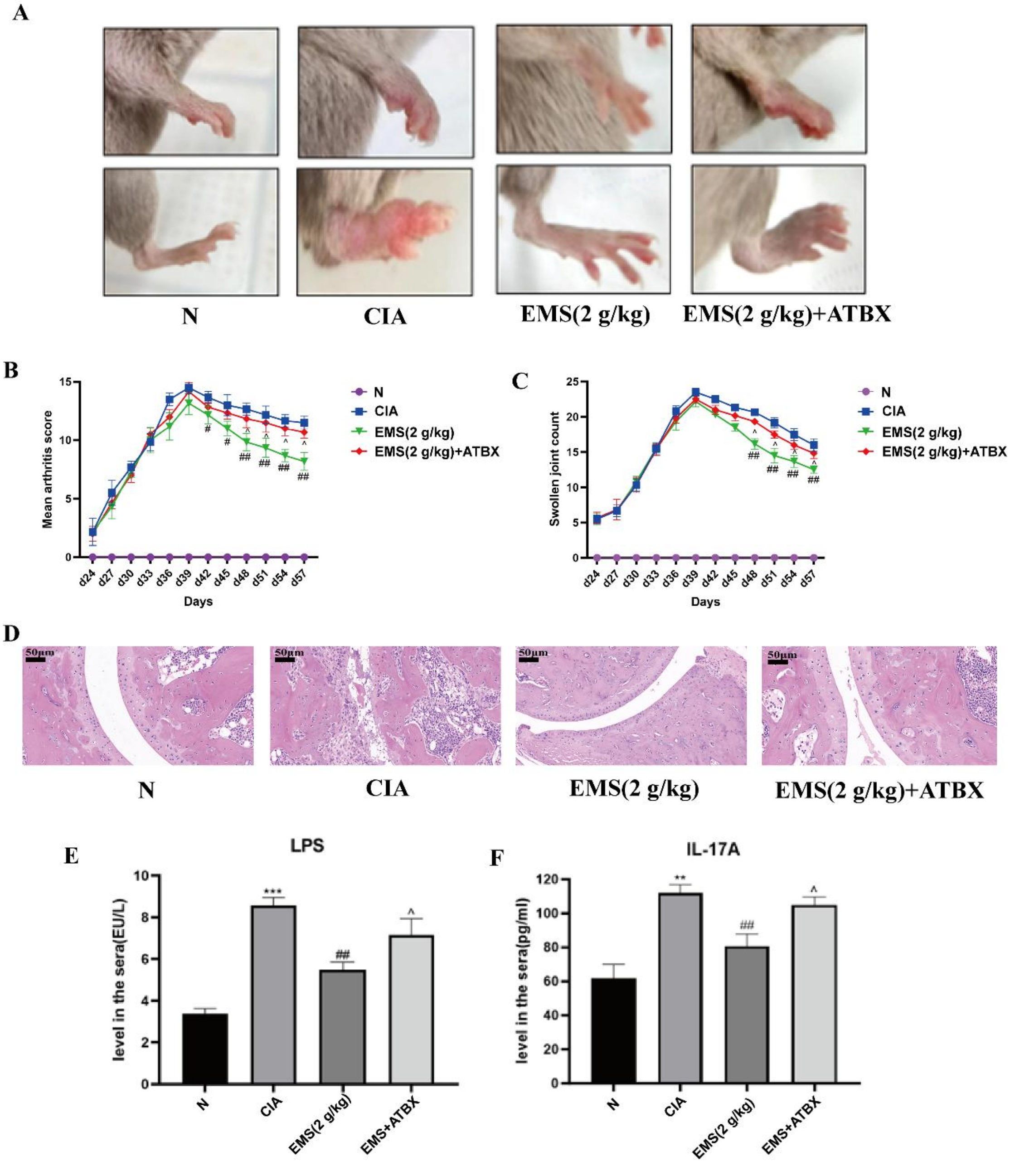

Fig. S2. ATBX attenuates the therapeutic effect of EMS on CIA mice.

(A) Representative plots of paw swelling in each group of mice. (n =6) **(B)** Arthritis index. (n = 6) **(C)** Swollen joint count. (n = 6) **(D)** HE staining of the ankle joint (HE, ×200 magnification) Scare bar: 50 μm. (n = 3) **(E-F)** The levels of IL-17A and LPS in the serum of CIA mice. (n = 4) Data are presented as the mean ± SD. $^{*}P$<0.05, $^{**}P$<0.01, $^{***}P$<0.001 vs. N group, $^{\#}P$<0.05, $^{\#\#}P$<0.01 vs. CIA group, $^{\wedge}P$<0.05 vs. EMS(2g/kg) group.
